# On the stability of the Bayenv method in assessing human SNP-environment associations

**DOI:** 10.1186/1479-7364-8-1

**Published:** 2014-01-09

**Authors:** Lily M Blair, Julie M Granka, Marcus W Feldman

**Affiliations:** 1 Department of Biology, Stanford University, Stanford, CA 94305, USA; 2 AncestryDNA, 153 Townsend St. Ste. 800, San Francisco, CA 94107, USA

**Keywords:** Environmental adaptation, Positive selection, Genome-wide scans, Human adaptation, Markov chain monte carlo, Natural selection

## Abstract

**Background:**

Phenotypic variation along environmental gradients has been documented among and within many species, and in some cases, genetic variation has been shown to be associated with these gradients. Bayenv is a relatively new method developed to detect patterns of polymorphisms associated with environmental gradients. Using a Bayesian Markov Chain Monte Carlo (MCMC) approach, Bayenv evaluates whether a linear model relating population allele frequencies to environmental variables is more probable than a null model based on observed frequencies of neutral markers. Although this method has been used to detect environmental adaptation in a number of species, including humans, plants, fish, and mosquitoes, stability between independent runs of this MCMC algorithm has not been characterized. In this paper, we explore the variability of results between runs and the factors contributing to it.

**Results:**

Independent runs of the Bayenv program were carried out using genome-wide single-nucleotide polymorphism (SNP) data from samples from 60 worldwide human populations following previous applications of the Bayenv method. To assess factors contributing to the method's stability, we used varying numbers of MCMC iterations and also analyzed a second modified data set that excluded two Siberian populations with extreme climate variables. Between any two runs, correlations between Bayes factors and the overlap of SNPs in the empirical *p* value tails were surprisingly low. Enrichments of genic versus non-genic SNPs in the empirical tails were more robust than the empirical *p* values; however, the significance of the enrichments for some environmental variables still varied among runs, contradicting previously published conclusions. Runs with a greater number of MCMC iterations slightly reduced run-to-run variability, and excluding the Siberian populations did not have a large effect on the stability of the runs.

**Conclusions:**

Because of high run-to-run variability, we advise against making conclusions about genome-wide patterns of adaptation based on only one run of the Bayenv algorithm and recommend caution in interpreting previous studies that have used only one run. Moving forward, we suggest carrying out multiple independent runs of Bayenv and averaging Bayes factors between runs to produce more stable and reliable results. With these modifications, future discoveries of environmental adaptation within species using the Bayenv method will be more accurate, interpretable, and easily compared between studies.

## Background

Phenotypic variation along environmental gradients, such as limb length in animals increasing with colder climates [1], animal body size increasing in colder environments [2], and bird coloration becoming darker in more humid climates [3], was first documented in the 1800s. Since then, many associations between phenotypic variation and environmental variation, both among and between species, have been noted [4-20]. To define these patterns, Huxley [21] introduced the term ‘cline’, which refers to a continuous phenotypic or genetic gradient that is associated with a pattern of geographic and/or environmental heterogeneity.

Various methods have been developed to determine the contribution of environmental variation to allelic or genotypic frequency patterns. Early theory in population genetics was used to derive statistics that allowed variation in allele frequencies to distinguish selection from drift [22-24]. Additionally, it has been shown that correlations between features of the environment and allele frequencies can be detected [15,25-27]. Recently, the availability of genome-wide data on DNA polymorphisms has spurred interest in using haplotype frequencies or allele frequency spectra to infer the existence or extent of selection across genomes [28-37].

Coop et al. [38] developed a statistical procedure named Bayenv to detect genomic evidence of adaptation to specific environmental variation, and it is this method we address here. The method carries out a locus-by-locus Bayesian analysis to determine whether a pattern in which population allele frequencies vary linearly with environmental variables is more probable than a pattern in which allele frequencies are assumed to be neutral and unrelated to environmental variables.

Given a set of populations with associated environmental variables and genome-wide single-nucleotide polymorphism (SNP) allele frequencies, a null model is first estimated to describe how allele frequencies covary across populations. Sample allele frequencies are drawn from a set of underlying population frequencies, which are assumed to be distributed according to a multivariate normal distribution around a transformed global allele frequency with a variance-covariance matrix representing the genetic structure of the populations. These transformed frequencies are not constrained between 0 and 1. The prior on the variance-covariance matrix is an inverse Wishart distribution, and Markov chain Monte Carlo (MCMC) computation is used to explore the posterior distribution of the covariance matrix, given the ancestral allele frequency and population allele frequencies.

After the covariance of allele frequencies between populations is estimated, the posterior probability of an alternative model for an individual SNP is determined. The alternative model predicts that the transformed population allele frequencies at a SNP are normally distributed around the ancestral allele frequency according to a linear model:

$$P\left(\theta |\Omega ,\alpha ,\beta \right)\sim N\left(\alpha +\beta Y,\alpha \left(1-\alpha \right)\Omega \right),$$

where *θ* is the transformed allele frequency, *α* is the ancestral allele frequency, *Y* is the environmental variable, and *Ω* is the variance-covariance matrix that was estimated in the first step of this procedure.

For each SNP, the Bayes factor (BF) is the posterior probability of the data at that SNP under the alternative model, integrated over all parameters, then divided by the posterior probability of the data under the null model, integrated over all parameters of the null model. Using a single run of the MCMC under the null distribution and importance sampling, the Bayes factors for multiple environmental variables are estimated quickly for each of the SNPs assayed (see additional details in Coop et al. [38]). This method has been shown to have good power compared to a range of other approaches [39].

The Bayenv method has been applied to many species including humans [40-44], plants [45-49], fish [50,51], and mosquitoes [52]. In all cases, the objective was to determine the extent to which a species' pattern of genomic variation can be inferred to be a response to environmental variation - for example climate, geography, photoperiod, or soil type - and hence could be regarded as a signal of adaptation.

Hancock et al. [40] applied the Bayenv method to genome-wide data from 61 worldwide populations for evidence of adaptation to 11 local climatic variables. Analyzing the pattern of variation of genome-wide SNP allele frequencies across the populations, they found evidence for adaptation to several variables, the most significant being latitude, summer relative humidity, summer solar radiation, and winter relative humidity. They reported signs of adaptation at SNPs previously identified by genome-wide association studies (GWAS) to be significantly related to phenotypes associated with environmental gradients, as well as an enrichment of genic versus non-genic SNPs among the SNPs most strongly correlated with the climate variables.

Although the Bayenv method has been applied to different species and environmental variables and has produced some results agreeing with other analytical methods, the stability of the results across multiple MCMC runs is not known. It is also unknown how the results may be affected by the inclusion of specific populations from extreme environments, which could potentially introduce bias [53]. In this study, we carried out many independent runs of the Bayenv program using data from Hancock et al. [40] to determine the degree of variability between runs and the factors that may contribute to any variability.

During the writing of this manuscript, an updated version of this program, Bayenv 2.0 [53], was released. While the Bayes factor estimation procedure is the same, it adds features that allow for the input of pooled population sequencing data and the computation of non-parametric correlations of the allele frequencies with the environmental variables. Although using this new version is advisable, the analyses of the original Bayenv program presented here remain relevant both to further applications of the method as well as to studies using results of previous Bayenv analyses (i.e., Fraser [54]).

## Results

### All 60 populations; 100,000 MCMC iterations

First, we analyzed the full data set of 60 populations and assessed correlations with 11 climate variables using 100,000 MCMC iterations (as in Hancock et al. [40]). We compared results between pairs of five independent runs as well as between each of our runs and Hancock et al.'s [40] published results. We report comparisons of our runs with an updated set of results made available online by the authors (which we refer to as Hancock2) that used a different number of MCMC burn-ins from the original set of results made available (Hancock1) (see ‘Methods’ section for details). The 16 pairs analyzed here are shown by a's in Table 1.

**Table 1:** Pairs of Bayenv runs compared

	**Hancock1**	**Hancock2**	**Blair1**	**Blair2**	**Blair3**	**Blair4**	**Blair5**	**LongBlair1**	**LongBlair2**	**LongBlair3**	**LongBlair4**	**LongBlair5**	**W/O_Sib1**	**W/O_Sib2**	**W/O_Sib3**	**W/O_Sib4**	**W/O_Sib5**
Hancock1																	
Hancock2	a																
Blair1		a															
Blair2		a	a														
Blair3		a	a	a													
Blair4		a	a	a	a												
Blair5		a	a	a	a	a											
LongBlair1																	
LongBlair2								b									
LongBlair3								b	b								
LongBlair4								b	b	b							
LongBlair5								b	b	b	b						
W/O_Sib1		d															
W/O_Sib2		d											c				
W/O_Sib3		d											c	c			
W/O_Sib4		d											c	c	c		
W/O_Sib5		d											c	c	c	c	

Letters indicate the parts of Tables 2 and 3 in which results for the indicated comparisons are averaged and presented. In Table 2, letters a, b, c, and d also correspond to parts e, f, g, and h, respectively. Hancock1 refers to the original Climate file posted on the dbCline website and published in Hancock et al. [40]; Hancock2 refers to file Climate.2 posted on dbCline website in autumn 2012 that uses the appropriate MCMC burn-in (see ‘Methods’ section); Blair1-5 refer to our five separate runs using 100,000 MCMC iterations; LongBlair1-5 refer to our five separate runs using 500,000 MCMC iterations; W/O_Sib1-5 refer to our five runs using a modified data set excluding the Siberian populations.

We found that Bayenv produced slightly different results in independent runs of the program. For a given climate variable, pairwise correlations of the logarithms of Bayes factors (BF's) were computed for all SNPs from each run, and correlations were averaged across all pairs of runs mentioned above (shown in Table 1). The averaged correlation coefficients ranged from 0.66 to 0.89 (Table 2a), depending on the climate variable. Despite analyzing only 60 of the 61 populations analyzed in Hancock et al. [40], correlations among our runs were similar to correlations with results from Hancock et al. [40]. Correlations between the two versions of Hancock et al.'s [40] published results were also comparable.

**Table 2:** **Average correlations of the log of the Bayes factors (BF) and average correlations of the empirical** ***p*** **values between runs for each climate variable**

	**Latitude**	**Absolute value latitude**	**Longitude**	**Min temp win**	**Max temp sum**	**Prec. rate sum**	**Prec. rate win**	**Rad flux sum**	**Rad flux win**	**Rel humid sum**	**Rel humid Win**
Log of BF											
(a) All populations (100,000 iterations) incl Hancock	0.87	0.88	0.66	0.83	0.84	0.73	0.75	0.74	0.89	0.73	0.79
(b) All populations (500,000 iterations)	0.93	0.92	0.82	0.88	0.90	0.85	0.85	0.83	0.93	0.85	0.88
(c) Without Siberia (150,000 iterations)	0.87	0.87	0.74	0.82	0.86	0.79	0.81	0.78	0.87	0.80	0.85
(d) Without Siberia (150,000 iterations) vs. Hancock2	0.79	0.82	0.70	0.81	0.81	0.70	0.72	0.75	0.84	0.78	0.76
Empirical *p* values											
(e) All populations (100,000 iterations) incl Hancock	0.81	0.84	0.63	0.79	0.81	0.68	0.70	0.70	0.85	0.70	0.75
(f) All populations (500,000 iterations)	0.91	0.91	0.81	0.88	0.90	0.82	0.83	0.82	0.92	0.84	0.86
(g) Without Siberia (150,000 iterations)	0.82	0.81	0.70	0.80	0.82	0.73	0.75	0.74	0.82	0.76	0.80
(h) Without Siberia (150,000 iterations) vs. Hancock2	0.61	0.65	0.67	0.74	0.66	0.62	0.68	0.70	0.67	0.72	0.75

‘Win’ refers to winter, ‘sum’ refers to summer, ‘prec. rate’ refers to precipitation rate, ‘rad. flux’ refers to radiation flux, and ‘rel. humid’ refers to relative humidity. Runs compared in each part are shown in Table 1. Incl Hancock means that the average is taken over pairs of Blair1-5 (100,000 iterations) and Hancock1-2, shown by a's in Table 1. Hancock1-2 were run with 150,000 iterations.

Correlations of empirical *p* values for all SNPs between the pairs of runs listed in Table 1 were also calculated; these correlation coefficients ranged from 0.63 to 0.85 (Table 2e), depending on the climate variable. Again, all pairs of runs compared displayed similar correlation values. For both the BF and *p* value correlations, the longitude variable had the lowest correlation among runs while winter radiation flux had the highest.

To examine the consistency of SNPs present in the empirical tails of each run, the overlap of SNPs in the tails with *p* value cutoffs of 0.05, 0.01, 0.005, and 0.001 was calculated. Overlap was defined as the proportion of overlapping SNPs relative to the total number of SNPs in the tail; this proportion was averaged over all climate variables. All pairs of runs showed comparable proportions of overlaps, both when compared among the Blair runs and between Blair runs and Hancock2. These overlap proportions ranged from 0.24 in the 0.001 tails of both runs to 0.57 in the 0.05 tails of both runs when Blair1 was compared to Hancock2 (Table 3a), with similar values for overlap between all Blair runs. In general, greater SNP overlap occurred when the SNPs in one run's tail were compared to the less extreme tail in another run. (In other words, more SNPs in the 0.001 tail of run A overlapped with the SNPs in the 0.005 tail of run B than the 0.001 tail of run B, and even more overlapped with the SNPs in the 0.01 tail of run B).

**Table 3:** **Empirical** ***p*** **value tail comparisons between indicated runs**

		**.05**	**.01**	**.005**	**.001**
(a) All populations (100,000 iterations) - Hancock2 vs. Blair1	**.05**	0.55	0.79	0.83	0.88
	**.01**	0.75	0.40	0.51	0.66
	**.005**	0.78	0.49	0.34	0.54
	**.001**	0.83	0.62	0.51	0.24
(b) All populations (500,000 iterations) - LongBlair1 vs. LongBlair3	**.05**	0.64	0.85	0.89	0.93
	**.01**	0.88	0.51	0.62	0.78
	**.005**	0.91	0.65	0.46	0.67
	**.001**	0.94	0.80	0.70	0.37
(c) Without Siberia (150,000 iterations) - W/O_Sib1 vs. W/O_Sib3	**.05**	0.60	0.83	0.86	0.92
	**.01**	0.81	0.45	0.56	0.73
	**.005**	0.85	0.56	0.40	0.61
	**.001**	0.90	0.71	0.59	0.30
(d) Without Siberia (150,000 iterations) vs. Hancock2 - Hancock2 vs. W/O_Sib1	**.05**	0.51	0.72	0.75	0.80
	**.01**	0.75	0.36	0.44	0.56
	**.005**	0.81	0.46	0.30	0.44
	**.001**	0.87	0.62	0.48	0.20

The table reports the fraction of SNPs from the smaller tail of one run that is present in the larger tail of the second run, averaged over climate variables. Two identical runs produce values of 1. Within each part (a to d), all pairs of runs (see Table 1) have similar fractions of overlap, and representative tables are shown here. For a given part, the first run's empirical tails are shown as rows (e.g., Hancock2 in part a), and the second run's are shown as columns (e.g., Blair1 in part a). Also, 623,318 SNPs are included in this analysis.

To assess whether SNPs with more extreme *p* values had greater overlap among runs, we ordered the empirical *p* values from the lowest to the highest for each climate variable and binned the SNPs in groups of 1,000. Each bin was compared to the corresponding ordered bin in each of the other Blair runs. Bins of lower *p* values had the highest overlap and overlap decreased as the empirical *p* values increased. The mean overlap for the top 1,000 SNPs was 0.44, and overlap was much lower for less significant SNPs (Table 4).

**Table 4:** **Overlap of ranked SNPs (ranked by empirical** ***p*** **value from lowest to highest and binned in groups of 1,000) between runs**

**Range of ranked SNPs**	**Overlap proportion**
1–1,000	0.44 ± 0.020
100,001–101,000	0.0073 ± 0.0033
200,001–201,000	0.0051 ± 0.0023
300,001–301,000	0.0043 ± 0.0016
400,001–401,000	0.0042 ± 0.0014
500,001–501,000	0.0035 ± 0.0014
600,001–601,000	0.014 ± 0.0098

For each bin, we calculated the proportion of overlapping SNPs between each corresponding bin for all ten pairs of runs (Blair1-5). Table reports the mean and standard deviation of the overlap proportion across all pairs of runs. Only 1 bin per 100,000 SNPs is shown. Also, 623,318 total SNPs were used in the analysis.

There was also variation among the results at individual SNPs. For example, SNPs in the CORIN gene region (rs4558846, rs6447571, rs17601068) gave strong signals (log_10_BF = 21.9, 28.7, and 20.8 and empirical *p* values = 2.0 × 10^-5^, 3.1 × 10^-5^, and 2.1 × 10^-5^) of association with minimum winter temperature as reported in Hancock et al. [40] (which we refer to as Hancock1), but show low signals in other runs: Blair1 (log_10_BF = -0.32, -0.50, and -0.48 and empirical *p* values = 0.40, 0.57, and 0.44) and Hancock2 (log_10_BF = -0.33, -0.07, and -0.16 and empirical *p* values = 0.36, 0.22, and 0.20). Some of this variability could be due to the limited burn-in in Hancock1 (see ‘Methods’ section). Other SNPs, such as rs2075756 in TRIP6, show more consistency. Blair1 obtained a log_10_BF of 4.0 and empirical *p* value of 7.9 × 10^-4^, while Hancock2 reported a log_10_BF of 1.4 and empirical *p* value of 3.2 × 10^-3^ for this SNP. This SNP shows a strong signal with absolute latitude, the variable for which we saw consistently strong signals of genic/non-genic enrichment among SNPs with low empirical *p* values (see below).

We then assessed whether genic SNPs were enriched among SNPs with the strongest signals by comparing the fraction of genic SNPs to non-genic SNPs in the empirical tails. This enrichment was compared across all five of our runs as well as with Hancock1 and Hancock2. The absolute enrichment values (calculated as described in the ‘Methods’ section) varied across runs and differed from Hancock's [40] published results (results not shown). The significance values of the genic/non-genic enrichments, which represent the proportion of bootstrapped samples that are also enriched, also varied across runs (i.e., for summer solar radiation) (Table 5).

**Table 5:** Genic/non-genic enrichments for each climate variable in the indicated empirical tails

**Climate variable**	**Data set**	**Empirical tail**		
		**0.05**	**0.01**	**0.005**
Absolute latitude	Hancock1 (published results)	3	3	3
	Hancock1 (our analysis)	1	2	2
	Hancock2	3	3	3
	Blair1	0	2	3
	Blair2	0	3	3
	Blair3	1	1	2
	Blair4	1	3	3
	Blair5	1	1	3
	LongBlair1	3	3	3
	LongBlair2	2	2	1
	LongBlair3	1	2	2
	LongBlair4	3	2	1
	LongBlair5	2	2	3
	W/O_Sib1	1	0	0
	W/O_Sib2	0	1	2
	W/O_Sib3	1	0	1
	W/O_Sib4	0	0	0
	W/O_Sib5	0	0	0
Summer maximum temperature	Hancock1 (published results)	0	0	2
	Hancock1 (our analysis)	0	2	3
	Hancock2	2	3	3
	Blair1	0	0	0
	Blair2	0	0	0
	Blair3	0	1	0
	Blair4	0	0	0
	Blair5	1	0	0
	LongBlair1	0	0	1
	LongBlair2	0	1	0
	LongBlair3	0	0	1
	LongBlair4	0	2	2
	LongBlair5	0	0	1
	W/O_Sib1	3	2	2
	W/O_Sib2	0	0	0
	W/O_Sib3	0	0	0
	W/O_Sib4	0	2	3
	W/O_Sib5	3	0	0
Summer precipitation rate	Hancock1 (published results)	0	0	0
	Hancock1 (our analysis)	0	0	0
	Hancock2	0	0	0
	Blair1	0	0	0
	Blair2	0	0	0
	Blair3	0	0	0
	Blair4	0	0	0
	Blair5	0	0	0
	LongBlair1	0	0	0
	LongBlair2	0	0	0
	LongBlair3	0	0	0
	LongBlair4	0	0	0
	LongBlair5	0	0	0
	W/O_Sib1	0	0	0
	W/O_Sib2	0	0	0
	W/O_Sib3	0	0	0
	W/O_Sib4	0	0	0
	W/O_Sib5	0	0	0
Summer solar radiation	Hancock1 (published results)	3	3	3
	Hancock1 (our analysis)	3	3	3
	Hancock2	0	1	3
	Blair1	2	2	0
	Blair2	0	0	0
	Blair3	1	0	0
	Blair4	0	0	0
	Blair5	3	0	0
	LongBlair1	2	3	3
	LongBlair2	0	3	3
	LongBlair3	2	0	0
	LongBlair4	0	2	1
	LongBlair5	0	3	3
	W/O_Sib1	3	3	3
	W/O_Sib2	1	0	0
	W/O_Sib3	0	0	0
	W/O_Sib4	1	0	0
	W/O_Sib5	3	3	2
Summer relative humidity	Hancock1 (published results)	2	3	3
	Hancock1 (our analysis)	2	3	3
	Hancock2	0	3	3
	Blair1	0	2	1
	Blair2	2	0	0
	Blair3	0	0	0
	Blair4	0	2	0
	Blair5	1	0	0
	LongBlair1	0	0	0
	LongBlair2	1	2	3
	LongBlair3	0	2	1
	LongBlair4	0	0	1
	LongBlair5	1	1	1
	W/O_Sib1	0	3	3
	W/O_Sib2	2	3	2
	W/O_Sib3	0	0	0
	W/O_Sib4	1	2	1
	W/O_Sib5	0	0	1
Winter minimum temperature	Hancock1 (published results)	1	0	0
	Hancock1 (our analysis)	1	0	0
	Hancock2	0	3	3
	Blair1	0	1	2
	Blair2	0	2	2
	Blair3	0	1	2
	Blair4	0	0	1
	Blair5	0	1	1
	LongBlair1	0	3	2
	LongBlair2	0	2	2
	LongBlair3	0	3	2
	LongBlair4	0	2	2
	LongBlair5	0	3	3
	W/O_Sib1	0	0	0
	W/O_Sib2	0	2	0
	W/O_Sib3	1	0	2
	W/O_Sib4	0	1	3
	W/O_Sib5	0	1	2
Winter precipitation rate	Hancock1 (published results)	0	3	3
	Hancock1 (our analysis)	1	3	3
	Hancock2	2	0	0
	Blair1	2	0	0
	Blair2	0	1	0
	Blair3	2	1	0
	Blair4	2	3	3
	Blair5	0	0	0
	LongBlair1	0	0	0
	LongBlair2	0	0	0
	LongBlair3	0	0	0
	LongBlair4	0	0	0
	LongBlair5	0	2	0
	W/O_Sib1	0	0	2
	W/O_Sib2	0	2	3
	W/O_Sib3	0	0	0
	W/O_Sib4	0	3	2
	W/O_Sib5	3	1	2
Winter relative humidity	Hancock1 (published results)	3	3	3
	Hancock1 (our analysis)	3	2	2
	Hancock2	3	3	3
	Blair1	3	3	3
	Blair2	3	3	2
	Blair3	3	1	3
	Blair4	3	3	3
	Blair5	3	1	0
	LongBlair1	3	3	0
	LongBlair2	3	3	2
	LongBlair3	3	3	2
	LongBlair4	2	0	0
	LongBlair5	3	2	1
	W/O_Sib1	3	2	2
	W/O_Sib2	3	2	3
	W/O_Sib3	3	3	2
	W/O_Sib4	3	3	3
	W/O_Sib5	3	3	0
Winter solar radiation	Hancock1 (published results)	3	1	1
	Hancock1 (our analysis)	3	0	2
	Hancock2	3	2	0
	Blair1	3	1	0
	Blair2	3	2	1
	Blair3	3	0	0
	Blair4	3	0	0
	Blair5	2	1	0
	LongBlair1	3	0	0
	LongBlair2	3	2	0
	LongBlair3	3	2	1
	LongBlair4	3	3	2
	LongBlair5	3	1	0
	W/O_Sib1	3	0	0
	W/O_Sib2	3	1	2
	W/O_Sib3	3	0	0
	W/O_Sib4	3	3	2
	W/O_Sib5	2	0	1

The table shows the number of stars of significance for genic/non-genic enrichments in the 0.05, 0.01, and 0.005 tails of the empirical *p* value distribution for each indicated run (defined as in Table 1 and in the ‘Methods’ section). ‘1’ denotes 95%–97.5% of bootstraps had an enrichment of genic compared to non-genic SNPs in that empirical tail, ‘2’ denotes 97.5%–99%, and ‘3’ denotes >99%. ‘0’ denotes that a genic enrichment is not significant (i.e., that <95% of the bootstraps had an enrichment). Enrichments for the nine environmental variables that were shown in Hancock et al. [40] are shown here.

The absolute latitude and winter relative humidity variables were almost always significant, with the exception of the 0.005 tail in Blair5; the summer precipitation variable was never significant. All three of these findings agreed with Hancock et al.'s published results [40]. However, summer solar radiation and humidity were not consistently significant, though reported to be significant in Hancock et al. [40]. Among the remaining variables, results varied among runs, and our runs showed less significance on average than Hancock et al.'s published results [40].

### All 60 populations; 500,000 MCMC iterations

In order to determine whether the observed variability between runs was due to the MCMC not converging, the number of MCMC iterations was increased fivefold to 500,000. Five independent runs were performed, each with different seeds, but with the same full data set of 60 populations. All pairs of the five runs with 500,000 iterations were compared (shown as b's in Table 1).

Between independent runs, both the Bayes factors and *p* values showed higher correlation coefficients compared to those between independent runs with 100,000 iterations. When averaged across all pairs of runs, the Bayes factor correlation coefficients ranged from 0.82 to 0.95 (Table 2b), and the correlation coefficients of the *p* values ranged from 0.81 to 0.92 (Table 2f). These correlations are significantly higher than among runs of 100,000 iterations for all environmental variables: paired Wilcoxon tests of these correlations, which for each climate variable are averaged across pairs of runs, give a *p* value of 0.0038 for the Bayes factors and a *p* value of 0.0038 for the empirical *p* values. The SNPs in the empirical *p* value tails were compared as before, and although the overlap was greater between long runs than between runs with 100,000 iterations, the proportion of overlapping SNPs in the tails was still not high, ranging from 0.64 in the 0.05 tails of two runs to 0.36 in the 0.001 tails of two runs (Table 3b).

Genic/non-genic enrichment significance values also stabilized somewhat in the runs with 500,000 iterations but still did not show the same significance values from run to run (Table 5). Genic/non-genic enrichments were highly significant for the absolute latitude and relative humidity winter variables and again were not significant for the summer precipitation variable across all five runs. Interestingly, none of the five runs showed significant genic/non-genic enrichment for winter precipitation rate, except in the 0.01 tail in Blair5. As before, varying levels of significance were seen for summer solar radiation and summer relative humidity. This differs from Hancock et al. [40], who reported high significance across all tails for these environmental variables.

In searching for the source of the differences described above, biases from allele frequency, SNP ascertainment panel, and method of calculating empirical *p* values were investigated, but none of these potential biases was shown to explain the variability among runs. First, the data were re-analyzed using empirical *p* values that were not binned by allele frequency. *P* value overlap did not increase when binned vs. un-binned *p* values were compared. Second, the proportions of SNPs from each ascertainment panel in each tail were calculated, and these proportions were found to be similar to the overall proportions of SNPs in each ascertainment panel. Additionally, when each ascertainment panel was considered separately, the overlap in the tails of *p* values was similar to the overlap in the tails when all three ascertainment panels were considered together.

### Without Siberian populations; 150,000 iterations

Because the Siberian populations, the Naukan Yup’ik and Maritime Chukchee, live in some of the most extreme climatic regions among the 60 sampled populations, it may be possible that removing these populations from the data set could affect the variability of Bayes factors, empirical *p* values, and genic/non-genic enrichments among independent runs. All pairs of runs using this modified data set were compared (shown as c's in Table 1). Pairwise correlations of the log of the Bayes factors (Table 2c) and *p* values (Table 2g) as well as fractions of overlap of SNPs in the 0.05, 0.01, 0.005, and 0.001 tails of the empirical *p* values (Table 3c) were comparable to the correlations between the runs of the full data set (60 populations with 100,000 iterations (Table 2a)). This suggests that the inclusion of populations with extreme environmental variables may not affect run-to-run variability.

To determine whether excluding the Siberian populations affected the results of the Bayenv method, each of the runs without the Siberian populations was compared to the updated Hancock results published on the dbCline website (Hancock2) (shown by d's in Table 1). Pairwise correlations between the log of the Bayes factors of these pairs (Table 2d), averaged across all pairs, were similar to correlations between pairs of non-Siberian runs (Table 2c). However, correlations of empirical *p* values between Hancock2 and non-Siberian runs (Table 2h) were lower than correlations between pairs of non-Siberian runs (Table 2g) and also lower than the correlations between pairs of runs using the full data set (Table 2e). Similarly, overlap in the empirical tails is lower when non-Siberian runs are compared to the results from Hancock2 (Table 3d), demonstrating a slight difference in results when these populations were excluded.

Although genic/non-genic enrichment significance values varied across runs of the non-Siberian data set (Table 5), some patterns differed from the results that included the full data set of 60 populations. The genic/non-genic enrichment for the absolute latitude variable was less significant than in runs analyzing all populations (Table 5), showing a small effect of exclusion of the Siberian populations. In addition, winter precipitation rate was sometimes significant in the non-Siberian runs; this variable was significant in Hancock et al.'s [40] published data but not in our runs with the full population set using 500,000 MCMC iterations.

## Discussion

We have explored the computational replicability of the Bayenv program [38] in determining evidence for positive selection in response to climatic variation. We used genome-wide SNP data from 60 worldwide human populations and 11 climate variables as in Hancock et al. [40] and carried out independent Bayenv runs with 100,000 and 500,000 MCMC iterations, as well as with 150,000 iterations while omitting Siberian populations with extreme climatic variables. In comparing pairs of our runs as well as each of our runs with the results previously published in Hancock et al. [40], results from the Bayenv method appear to be variable across runs in their Bayes factors, empirical *p* values, and in the values and significances of enrichments of genic SNPs in the empirical tails. Although some run-to-run variability is expected in any MCMC algorithm, we show here that the differences between runs are sufficient to lead to varying biological conclusions regarding human adaptation.

In runs with 100,000 MCMC iterations, correlations of Bayes factors between replicate runs were low (0.66–0.88) between pairs of our runs, between our runs and the results of Hancock et al. [40], and between the different versions of results posted by Hancock et al. (Table 2a). To explore the possibility that the MCMC had failed to converge, we increased the number of MCMC iterations to 500,000; although these correlations increased as a result (0.82–0.93) (Table 2b), there was still substantial run-to-run variability in further analyses.

Because Bayes factors are dependent on the accuracy of the null model and, therefore, may be substantially inflated due to imperfections of this model [40], we focused further analyses on the empirical *p* values. Hancock et al. [40] write, ‘Since we cannot expect the null model to account fully for the effects of population structure, we emphasize that we cannot take the BFs themselves at face value, nor can they be directly compared across climate variables. Therefore, we took a conservative approach and conducted subsequent analyses by comparing each SNP to the empirical distribution.’ In our analysis, empirical *p* values were seen to be no more stable than the Bayes factors themselves, as correlation coefficients of the empirical *p* values were in the same range as the correlation coefficients of the Bayes factors (Table 2e). As before, the correlation of *p* values between runs improved when the MCMC iterations were increased to 500,000 (Table 2f). Although we recommend using a large number of MCMC iterations, this does not completely stabilize Bayenv results from run to run.

Amounts of SNP overlap in the tails of the empirical *p* value distributions showed that the relative ranking of the SNPs associated with environmental variables varied across runs, even with a larger number of MCMC iterations. While the overlap of SNPs between the extreme tails (i.e., top 0.01%) of two runs was found to be low, we recognize that this slightly overestimates run-to-run variability. For example, 24% of the 0.001 tail of Hancock2 overlapped with the 0.001 tail of Blair1; that proportion increased to 83% with the 0.005 tail of Blair1 (Table 3a). This suggests that the SNPs with the lowest empirical *p* values have generally low *p* values in all runs. Analysis of the overlap of SNPs with low empirical *p* values versus the overlap of SNPs with high or midrange empirical *p* values shows that strong signals are also more reproducible from run-to-run than weaker signals.

Despite this trend, a SNP's significance and relative ranking nearly always vary from one run of Bayenv to another. SNPs inferred to be significant using some pre-defined *p*-value threshold in one run may not be found to be significant in another run. Thus, the low fractions of empirical *p*-value tail overlap between runs suggest that caution is advisable in drawing inferences based on only one run, especially when making conclusions regarding individual SNPs or genes (see ‘Results’ section).

Finally, we assessed the stability of inferences regarding the enrichment of genic SNPs among SNPs strongly associated with a given climate variable. Evidence of such an enrichment has been argued as evidence of strong selection on genic regions in response to an associated environmental pressure [40,45]. Because the stability of these significances depends only on the ratio of genic to non-genic SNPs in the empirical tails, as opposed to the rank of each individual SNP, this measure could be consistent despite variability of Bayes factors and empirical *p* values. However, the significances of climate variables including summer maximum temperature, summer relative humidity, summer solar radiation, winter minimum temperature, winter precipitation rate, and winter solar radiation varied widely from run to run (Table 5).

Of particular interest are summer solar radiation and summer relative humidity, both claimed to be significant by Hancock et al. [40] but for which significance did not hold in our analyses. For these variables, as well as others, neither the enrichment values nor the significance of the enrichment values increased or decreased systematically when the tail cut-off was more extreme, as suggested in Hancock et al. [40]. The lack of significance for these variables in the Hancock2 analysis, which includes all 61 populations, shows that the exclusion of the data from Australian Aborigines from our analysis is not the cause for this difference in significance. Thus, replicate runs demonstrate that these two variables may have been less crucial in human adaptation than suggested previously [40].

Despite this variability between runs, certain climate variables show some consistency in genic/non-genic enrichments and agree with conclusions of Hancock et al. [40] (Table 5). Winter relative humidity is significant across all runs, including those in which the Siberian populations are excluded, suggesting that this may be a robust signal of adaptation in response to this climatic variable. Absolute latitude also shows a strong signal and is significant across all runs that include all populations. That we were able to replicate the significance of both of these variables, despite the fact that we did not include the Australian Aborigines (included in Hancock et al. [40]) underscores their potential importance in human adaptation.

We note that populations with extreme climate variables may indeed have an effect on some conclusions drawn from Bayenv analyses. For example, without the Siberian populations, an enrichment of genic SNPs was no longer significant for latitude (Table 5). Thus, while the inclusion of outlying populations may affect biological conclusions, it does not appear to affect run-to-run variability (see ‘Results’ section).

In summary, our analysis of the consistency of genic enrichments between runs demonstrates the importance of performing multiple runs of Bayenv. Two independent runs can potentially lead to very different biological conclusions. Significance of a particular environmental variable in multiple Bayenv runs constitutes much stronger evidence for its relevance and importance for human adaptation.

A new version of the Bayenv method, Bayenv 2.0 [53], has recently been released. Running the new version with 100,000 MCMC iterations produces slightly more stable results among runs than the old version; however, the variability is comparable to the runs of the old version with 500,000 MCMC iterations (results not shown). Gunther et al. note that their inclusion of non-parametric analyses in Bayenv 2.0 helps to account for potential false positives due to outlying populations [53]; however, our results suggest that results run on the previous version of the Bayenv program that do *not* include outlying populations may still lead to varying results across runs. Thus, while we suggest using the new version, the results described in this manuscript remain relevant to the application of Bayenv 2.0.

It can be difficult to disentangle stability and specificity, and we recognize that some of the run-to-run variability of this method may be due to less strong signals of environmental adaptation (i.e., winter minimum temperature as compared to latitude). However, as every run may be different, it is still advisable to carry out multiple runs when making biological conclusions, especially when the signal tested may not be strong to begin with. Averaging Bayes factors of two or more independent runs before calculating the empirical *p* values produces more stable empirical *p* values and genic/non-genic enrichments (results not shown). Additionally, individual SNPs that show large Bayes factors in at least one run could be rerun with more MCMC iterations to determine a Bayes factor value to which all iterations converge. Unfortunately, this process would not be as useful for a genome-wide analysis, as a large set of SNPs is necessary for the calculation of the empirical *p* values and the genic/non-genic enrichments.

## Conclusions

We conclude that, in using the Bayenv method, relying on the relative rankings of SNPs calculated from only one run of the program may lead to biological conclusions that might not have been reached with another independent run of the program. Neither increasing the number of MCMC iterations nor removing outlying populations completely eliminated this variability. Thus, we suggest carrying out several independent runs of the Bayenv analysis to assess variability of the Bayes factors and empirical *p* values across runs. We also suggest averaging Bayes factors from independent runs to produce more stable results. With these modifications, future discoveries of environmental adaptation within species using the Bayenv method will be more accurate, interpretable, and easily compared between studies.

## Methods

### Populations

Allele frequency data on 623,318 SNPs from samples of 60 human populations from the freq_data file (61pops_climate_freqs.txt) on the dbCline website ( http://genapps2.uchicago.edu:8081/dbcline/main.jsp) were analyzed. The data set included samples from 52 HGDP populations plus Vasekela !Kung, Amhara, Naukan Yup’ik, Maritime Chukchee, Luhya, Maasai, Tuscans, and Gujarati. These were the same populations used by Hancock et al. [40], with the exception of the Australian Aborigines, who were not included in the allele frequency file on dbCline. To translate allele frequencies into allele counts, the sample sizes from Li et al. [55] were used for the HGDP populations and from the supplementary information of Hancock et al. [40] for the additional eight populations.

### Climate variables

The climate data consisted of the 11 variables in Hancock et al. [40] that are posted on the dbCline website: latitude, absolute latitude, longitude, winter minimum temperature, summer maximum temperature, summer precipitation rate, winter precipitation rate, summer radiation flux, winter radiation flux, summer relative humidity, and winter relative humidity. Climate data were obtained for each population directly from the dbCline website (61pops_climate_freqs.txt).

### Covariance matrix estimation

For the set of 60 populations, three covariance matrices, each one representing a different ascertainment panel (Illumina 250 K, Illumina 300 K, or Illumina AFR), were generated using the Bayenv program (as in Hancock et al. [40]). The program was run for 100,000 MCMC iterations, and the entries of each matrix were averaged over the last three output matrices (output every 5,000 iterations). Visual comparison showed the matrices to be qualitatively similar to those in Coop et al. [38], and the matrices were stable from run to run. For our analysis, one covariance matrix for each ascertainment panel was estimated as described here and used for all runs.

### Bayenv runs

Five independent runs of the Bayenv program were carried out using the full data set of 60 populations and 11 climate variables. As with the covariance matrices, SNPs were analyzed with their corresponding ascertainment panel. Each run used a different random seed but the same input data set and 100,000 MCMC iterations. Bayes factors produced from the three ascertainment panels were combined into one file for each run. These runs are referred to as Blair1 - Blair5 (see Table 1).

In a separate analysis, five independent runs of the Bayenv program were carried out as above, using the same full data set but increasing the MCMC iterations to 500,000 (referred to as LongBlair1 - LongBlair5) (see Table 1). For all runs, we use the default MCMC burn-in values.

### Bayenv runs without Siberia

Five independent runs of the Bayenv program were carried out using a modified data set and 150,000 MCMC iterations. In this modified data set, all 11 climate variables were included, but the Siberian populations (Naukan Yup’ik and Maritime Chukchee) were excluded (referred to as W/O Siberia1 - W/O Siberia5; see Table 1). The aim here was to explore how the Siberian populations, who live in some of the most extreme climatic regions, might affect the stability and conclusions drawn from Bayes factors, empirical *p* values, and genic/non-genic enrichment values (see below).

### Hancock runs

Bayes factors and empirical *p* values from Hancock et al. [40] were obtained from the dbCline website. Following the Hancock et al. [40] publication, the authors posted a set of results, which we refer to as Hancock1, which used fewer burn-ins for the MCMC procedure and thus failed to converge (Anna Di Rienzo, personal correspondence). After further correspondence with the authors, a second set of results that used a longer burn-in was posted in autumn 2013 (which we refer to as Hancock2). We examined BF correlations and empirical *p* value correlations and overlap between these two data sets (described below). For calculations of genic/non-genic enrichment (described below), we performed the analysis on both Hancock1 and Hancock2. In general, our analysis of the Hancock1 data set gave similar results to those published by Hancock et al. [40].

### SNP *p* values

As in Hancock et al. [40], we calculated empirical *p* values by binning SNPs based on their global allele frequency (across 60 populations). For each SNP, a transformed rank statistic was computed by comparing the computed Bayes factor for the SNP with the Bayes factors from other SNPs in the same global allele frequency bin. For this process, all SNPs were analyzed together, and no distinction was made for SNPs belonging to different ascertainment panels.

### Genic/non-genic enrichments

To calculate genic/non-genic enrichments, a SNP was defined as genic if it was within 10 kb of a gene and non-genic if it was greater than 50 kb from a gene (as in Hancock et al. [40], personal correspondence). Enrichment in an empirical tail was calculated with the following equation:

$$\text{Enrichment}=\frac{\frac{n_{g}}{n_{\text{ng}}}}{\frac{N_{g}}{N_{\text{ng}}}},$$

where *n*_g_ and *n*_ng_ are the number of genic and non-genic SNPs, respectively, in the empirical tail, and *N*_g_ and *N*_ng_ are the number of genic and non-genic SNPs, respectively, among all tested SNPs. To determine the significance of the genic/non-genic enrichments, 500-kb regions from the genome were re-sampled with replacement (also as in Hancock et al. [40]). Then, given the *p* values of the SNPs in the re-sampled regions, we calculated enrichment values in each empirical tail. This bootstrap re-sampling procedure was repeated 10,000 times. Significance values were determined by counting the fraction of bootstrapped samples with enrichment values above 1. In Table 5, one star denotes that 95%–97.5% of bootstrapped samples were enriched, two stars denote 97.5%–99%, and three stars denote >99%; zero stars denotes that <95% of bootstrap samples were enriched, and thus that the genic enrichment is not significant.

### Comparison of runs

Each run produces a Bayes factor for each climate variable for each SNP. From those, the empirical *p* values were calculated. For each climate variable, the Pearson correlations of the log of the Bayes factors and the Pearson correlations of the empirical *p* values were computed for each pair of runs compared (see Table 1). For each set of comparisons (i.e., Blair1-5, or W/O_Sib1-5 vs. Hancock2) the correlation coefficients were then averaged across all relevant pairs of runs (see letters of Table 1). For all pairs shown in Table 1, empirical *p* values were also compared by calculating the fraction of SNPs in the tail of one run that was present in the tail of the other run. In another analysis, to assess differences in run stability by empirical *p* value, SNPs were ordered by empirical *p* value from lowest to highest and binned into groups of 1,000. Overlaps between the corresponding bins of different runs (Blair1-5) were calculated. (This assessed what proportion of the 1,000 most-significant SNPs in Blair1 were present in the 1,000 most-significant SNPs in Blair2, what proportion of SNPs ranked 1,001–2,000 in Blair1 were present in SNPs ranked 1,001–2,000 of Blair2, and so forth.) Finally, significances of genic/non-genic enrichment values were calculated for each run, as described above, and compared.

## Abbreviations

BF: Bayes factor; MCMC: Markov Chain Monte Carlo; SNP: Single-nucleotide polymorphism.

## Competing interests

The authors declare that they have no competing interests.

## Authors' contributions

LB participated in the design of the study and performed the statistical analysis. JG and MF participated in its design and coordination. All authors read and approved the final manuscript.
